# *Plasmodium falciparum* Malaria in Children Aged 0-2 Years: The Role of Foetal Haemoglobin and Maternal Antibodies to Two Asexual Malaria Vaccine Candidates (MSP3 and GLURP)

**DOI:** 10.1371/journal.pone.0107965

**Published:** 2014-09-19

**Authors:** David Tiga Kangoye, Issa Nebie, Jean-Baptiste Yaro, Siaka Debe, Safiatou Traore, Oumarou Ouedraogo, Guillaume Sanou, Issiaka Soulama, Amidou Diarra, Alfred Tiono, Kevin Marsh, Sodiomon Bienvenu Sirima, Philip Bejon

**Affiliations:** 1 Centre National de Recherche et de Formation sur le Paludisme, Ouagadougou, Burkina Faso; 2 Kenyan Medical Research Institute, Centre for Geographic Medicine Research (Coast), Kilifi, Kenya; 3 Nuffield Department of Medicine, Centre for Clinical Vaccinology and Tropical Medicine, University of Oxford, Churchill Hospital, Oxford, United Kingdom; Universidade Federal de Minas Gerais, Brazil

## Abstract

**Background:**

Children below six months are reported to be less susceptible to clinical malaria. Maternally derived antibodies and foetal haemoglobin are important putative protective factors. We examined antibodies to *Plasmodium falciparum* merozoite surface protein 3 (MSP3) and glutamate-rich protein (GLURP), in children in their first two years of life in Burkina Faso and their risk of malaria.

**Methods:**

A cohort of 140 infants aged between four and six weeks was recruited in a stable transmission area of south-western Burkina Faso and monitored for 24 months by active and passive surveillance. Malaria infections were detected by examining blood smears using light microscopy. Enzyme-linked immunosorbent assay was used to quantify total Immunoglobulin G to *Plasmodium falciparum* antigens MSP3 and two regions of GLURP (R0 and R2) on blood samples collected at baseline, three, six, nine, 12, 18 and 24 months. Foetal haemoglobin and variant haemoglobin fractions were measured at the baseline visit using high pressure liquid chromatography.

**Results:**

A total of 79.6% of children experienced one or more episodes of febrile malaria during monitoring. Antibody titres to MSP3 were prospectively associated with an increased risk of malaria while antibody responses to GLURP (R0 and R2) did not alter the risk. Antibody titres to MSP3 were higher among children in areas of high malaria risk. Foetal haemoglobin was associated with delayed first episode of febrile malaria and haemoglobin CC type was associated with reduced incidence of febrile malaria.

**Conclusions:**

We did not find any evidence of association between titres of antibodies to MSP3, GLURP-R0 or GLURP-R2 as measured by enzyme-linked immunosorbent assay and early protection against malaria, although anti-MSP3 antibody titres may reflect increased exposure to malaria and therefore greater risk. Foetal haemoglobin was associated with protection against febrile malaria despite the study limitations and its role is therefore worthy further investigation.

## Introduction

Children under five years of age bear the majority of the malaria burden [1], however infants have been shown to be less likely to develop clinical malaria within their first six months of life [2]–[5].

This apparent protection has been linked to passive transfer of anti-malaria antibodies by earlier experimental studies [6]–[8]. A study in older children confirmed the protective effect of passive transfer of antibodies [9]. Other possible protective factors include foetal haemoglobin, as suggested by in vitro studies [10], [11] and mouse models [12]. Rodent malaria experiments have provided evidence of the protective effect of a para-aminobenzoic acid-poor diet [13]–[16], which can be expected in exclusive breast-feeding. Breast milk constituents might also contribute to the protection of infants against malaria as suggested by in vitro studies [17]. Finally, studies also suggested that infants are less exposed to mosquito bites [18], thus reducing their risk of contracting malaria.

Correlates of protection of specific antibodies against malaria have been extensively studied in adults and children [19]–[32], however fewer studies have specifically addressed the risk of malaria in infancy. Anti-malaria antibodies present during infancy are initially passively received from the mother in utero (mainly IgG) [33], and then endogenously produced by the infant (IgM and IgG) as a response to repeated exposure to infective mosquito bites [34]. Nonetheless, it has been evidenced that earlier in life, some foetuses are able to mount an immune response to prenatal exposure [35] through transplacental transfer of soluble malaria antigens [35], [36]. Most sero-epidemiological studies investigating these potential immune correlates of protection in infants did not find the hypothesised protective effect of antibodies to the individual antigens tested (CSP, LSA-1, crude schizont extract, MSP1, MSP2, Pf155/RESA and the vaccine candidate SPf66) [37]–[41]. However, two studies conducted independently in Liberia and Kenya [42], [43] identified a protective effect of antibodies to MSP1–19.

We performed the present longitudinal prospective infant cohort study in the south-western region of Burkina Faso, where the transmission of malaria is high and stable. Our study aimed at investigating the role of transplacentally acquired anti-malaria antibodies, while adjusting for foetal haemoglobin and other factors, in the susceptibility of children to *P. falciparum* malaria in their first years of life. We specifically investigated three merozoite surface proteins, MSP3-LSP and two regions of GLURP (R0 and R2), as they have been used in a malaria vaccine that is currently undergoing testing [44], [45].

## Methods

### Ethical consideration

This study was approved by the Institutional Review Board of Centre National de Recherche et de Formation sur le Paludisme (CNRFP) in Burkina Faso. The study was conducted according to the principles of the Declaration of Helsinki. Individual written informed consent was obtained from the parents of each child before any study procedure was performed. The parents who could not read and write in the language used in the informed consent form, signed the form with their thumb print after it was completed by an independent witness on their behalf. The IRB approved this consent procedure.

### Study site

The study area is described elsewhere [46]. Briefly, the study area encompasses four health catchment areas served by four dispensaries reporting to Banfora health district. Two (Flantama and Korona) are located within Banfora town with water and power supply and mainly cement brick, iron sheet roofing houses. The other two (Nafona and Bounouna) are Banfora sub-urban villages with mainly adobe-walled, thatched roofing houses. The annual rainfall is above 900 mm with the rains lasting from May to October. The malaria incidence in children under five is 1.18 episodes/child/year using an active case detection [46] with *Plasmodium falciparum* being responsible for more than 90% malaria cases.

### Study population

A cohort of 140 infants aged between four and six weeks was recruited into the study. The parents were informed of the study aims and procedures during the early post-natal visits at the dispensaries, prior to the children reaching one month of age. Recruitment was carried out simultaneously at the four health catchment areas of the study site from November 2010 to February 2011. Since measuring time to the first infection was one of the objectives of the study, infants who had either a documented previous episode of malaria or a positive blood smear at the baseline visit were excluded from the study; nevertheless, some infants might have had malaria infections that were unobserved. After the recruitment of the study participants, the geodetic coordinates (longitude, latitude) of their homesteads were recorded using Global Positioning System (GPS) devices. The approximate centre of each family compound was the reference point to record these coordinates.

### Surveillance of malaria morbidity

To detect malaria infections, the children were followed up actively with weekly home visits and passively with dispensary monitoring for two years. The active follow-up consisted of weekly home visits performed by fieldworkers whose main tasks were to check the children’s health, collect blood samples and start anti-malarial treatment when indicated according to the National Malaria Control Program guidelines. The time window for the home visits was ±2 days. In the passive follow-up, the caregivers were encouraged to bring their children to the nearby dispensary or the clinical research unit at any time should the child appear unwell. In both surveillance methods a blood smear was collected in case of fever (i.e. a reported history of fever in the past 24 hours and/or axillary temperature ≥37.5°C). In addition, to monitor the occurrence of asymptomatic parasitaemia, bloods smears were systematically collected on a monthly basis until the detection of the first malaria infection regardless of the axillary temperature. The children who became ill over the course of the study received health care free of charge either at the dispensaries, the clinical research unit or the paediatric unit of the regional referral hospital when necessary.

### Parasitological examination

Light microscopy was used to examine the blood smears, which were collected, air dried and GIEMSA-stained as described elsewhere [46]. The parasite density of each blood smear was assessed by two independent microscopists and their results were compared for consistency. When their results were concordant, the average was recorded as the final result; otherwise a third microscopist was involved. The final result was the average of the two most concordant parasite densities.

### Haemoglobin typing

High Pressure Liquid Chromatography (HPLC) was used to quantify the fractions of foetal haemoglobin and haemoglobin variants in the children.

### Antibody quantification

Enzyme-linked immunosorbent assay (ELISA) was used to quantify anti-malaria total IgG in capillary blood samples collected by finger or heel prick at baseline, three, six, nine, 12, 18 and 24 months of follow up. Two *P. falciparum* blood-stage antigens MSP3-LSP [47], GLURP (R0 and R2) [48] were used in these assays. The ELISA was performed as described elsewhere [24].

### Statistical analysis

Fever was defined as an axillary temperature ≥37.5°C and/or a reported history of fever in the past 24 hours. Malaria infection was defined as any positive parasitaemia regardless of the axillary temperature. Two definitions were set for febrile malaria; definition 1 included all febrile episodes with any level of asexual *P*. *falciparum* parasitaemia, and definition 2 included only febrile episodes associated with asexual *P. falciparum* parasite density ≥10000/µL. This latter definition was derived after examining the distributions of the log-transformed parasite densities among children with and without fever in cross-sectional surveys (Figure S1). Febrile malaria episodes occurring within 21 days were considered a single episode.

The antibody titres expressed in arbitrary units were log-transformed to approximate a normal distribution. We fitted a multiple fractional polynomial regression of antibody titres on age to estimate the non-linear relationship between anti-malaria antibody titres and age. We fitted a linear regression model to estimate the relationship between a set of potential predictors and anti-malaria antibody titres. In this linear model, antibody titre was included as a time-changing outcome, i.e. the antibody titre measured at the end of each time interval within which malaria infections were recorded, and age as fractional polynomial covariate.

We adapted a previously published method for calculating exposure indexes (EI) to our cohort with time-to-event data [49]. An individual malaria exposure index (EI) was computed as the median time to the first malaria infection of the surrounding neighbours of each index child within a circle of a given radius around him/her at the middle of the circle. We found that a 1.5 km radius best predicted risk in our dataset (Figure S2). EIs were transformed to negative values so that the most exposed has the highest exposure index.

We fitted a Cox regression model to estimate the relationship between the time to first febrile malaria episode and a set of covariates of interest. We also fitted a negative binomial regression model to estimate the association between multiple malaria episodes and a set of potential explanatory covariates. Antibodies were fit in two ways; a) applying the baseline antibody titre throughout the period of monitoring and b) applying time-varying antibody titre, i.e. the antibody titre measured at the most recent time point, which therefore changed throughout the period of monitoring. The log likelihood ratio test was used to test the significance of variables with multiple levels. The assumption of proportional hazards for Cox regression was tested based on the Kaplan Meier method and the Schoenfeld residuals. The assumption of a linear effect of antibody titres on malaria episodes was investigated using fractional polynomial modelling. We used the Huber-White Sandwich estimator to adjust for clustering by individual in linear and negative binomial regression models. The data were analysed using Stata 13 (StataCorp, College Station, Texas).

## Results

### Study population characteristics

A total of 216 mothers of new-borns were invited to attend study screening visits during early post-natal visits at the four dispensaries of the study area. Of these, 148 (68.5%) attended the study screening visits with their infants. A total of 140 infants were recruited during a three- month period from mid-November 2010 to mid-February 2011. The baseline characteristics of the infants and their mothers are summarised in Table 1.

**Table 1:** Baseline characteristics of the study population.

Characteristic	Statistic
Male_freq(%)	67 (47.9%)
Age infants(days)_median [min, max]	33 [27], [42]
Weight (kg)_ median [min, max]^1^	4.1 [2.8, 5.9]
Length (cm)_ median [min, max]^1^	54 [48, 61]
MUAC (cm)_ median [min, max]	12 [8.5, 16]
Hb rate (g/dL)_ median [min, max]	12.6 [8.7, 17.6]
Foetal Hb (%)_median [min, max]^3^	59.6 [20.1, 89.6]
Hb phenotype_freq (%)^3^	
AA	113 (80.71)
AC	18 (13.14)
AS	1 (0.73)
CC	5 (3.65)
Delivery way^2^	
Natural	133 (95)
Ceasarian section	5 (3.6)
Neonatal rescucitation_freq (%)	12 (8.6)
Neonatal infection_freq (%)	2 (1.43)
EPI (up to date at 1 month)_freq (%)	124 (88.6)
Age groups mothers (years)_freq (%)	
≤19	10 (7.1)
20–29	89 (63.6)
≥30	37 (26.4)
ITN use (during pregnancy)_freq (%)^1^	123 (87.9)
IPTp courses_freq (%)	
0	10 (7.14)
1	28 (20)
2	100 (71.43)
3	2 (1.43)
Gravidity status^2^ _freq (%)	
primigravidae	32 (22.86)
multigravidae	106 (75.71)
Education level (mothers)^1^_freq (%)	
No formal education	78 (55.71)
Primary school	39 (27.86)
Secondary school or above	22 (15.71)
Distribution of study population	
Bounouna	34 (22.97)
Nafona	41 (27.7)
Korona	16 (10.81)
Flantama	49 (33.11)

1, 2, 3 Number of missing data.

### Follow-up of participants and malaria morbidity

Twenty-three children (16.4%) were lost to follow-up before completing 24 months with a median [Inter Quartile Range-IQR] follow up time of 9.8 [2, 14.75] months. Among them, six migrated out of the study area, 10 withdrew their consent, four died and three dropped out of the study and were no longer reachable.

One or more episodes of febrile malaria were experienced by 79.6% of all children during follow-up. Sixty-three children (45.98%) had at least one malaria infection in their first year of life and 46 (76.66%) of the remaining 60 children in their second year.

### Anti-malaria antibody kinetics

Antibody titres from one time point were weakly to moderately correlated with antibodies at the next time point (rho ranging from –0.28 to 0.55; 0.31 to 0.58 and 0.15 to 0.60, respectively for anti-MSP3, anti-GLURP R0 and anti-GLURP R2 antibody titres) with the strongest correlations observed from baseline to month 3, and months 9 to 12 (Table S1). The time-course of individual antibody kinetics is shown in Figure S3. Anti-GLURP R0 and anti-GLURP R2 antibodies were more closely correlated to each other (r = 0.52, *p*<0.001) than either GLURP sub-unit antibody was correlated to anti-MSP3 antibody titres (r = 0.35, *p*<0.001 and r = 0.4, *p*<0.001 respectively).

**Figure 1:**
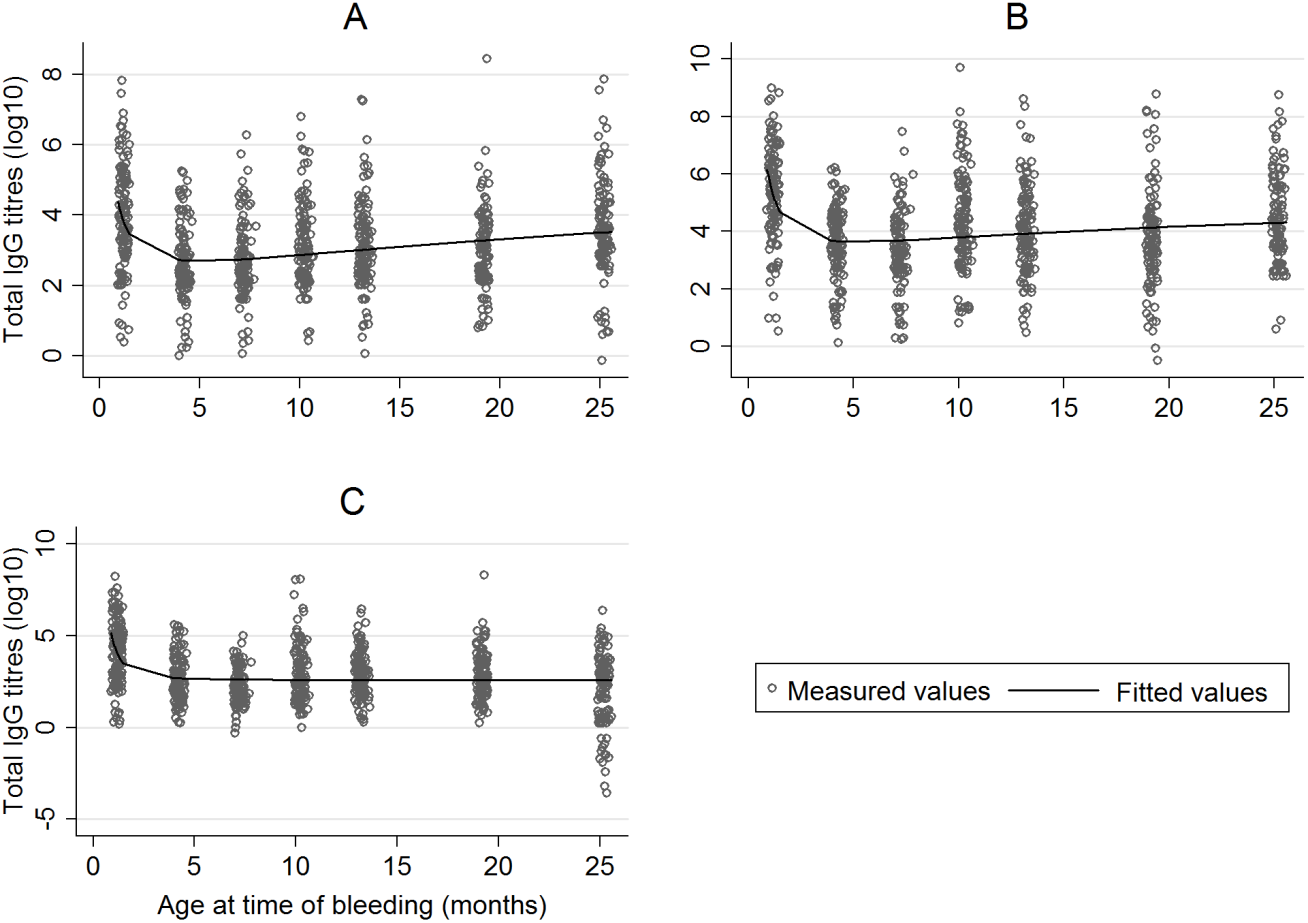
Antibody kinetics trends over the 24 months of observation. (A) Antibodies to GLURP R0, (B) Antibodies to GLURP R2, (C) Antibodies to MSP3.

Figure 1 shows individual antibody titres and a best-fit line using multiple fractional polynomials. There is an overall decline of anti-malaria total IgG titres to the three antigens from one to four months of age, presumably indicating the waning of maternally-derived anti-malaria antibodies. Thereafter, both the anti-GLURP R2 and anti-GLURP R0 total IgG titres rise slightly with increasing age while anti-MSP3 total IgG titres remain constant.

### Predictive factors for the changing antibody titres

The univariate analysis of all potential covariates is displayed in Table S2. The final multi-variable model showed strong associations with age and season of measurement for each antigen tested (Table 2). In addition, associations were observed between the number of malaria episodes recorded immediately before the blood sample collection and the titres of antibodies to GLURP.

**Table 2:** Multivariable predictive model for changing anti-malaria antibody titres.

	IgG anti-MSP3			IgG anti-GLURP R0			IgG anti-GLURP R2		
Predictor	Coef.	95% CI	*p*	Coef.	95% CI	*p*	Coef.	95% CI	*p*
Age power(−2/−5/−5)^a^	0.02	[0.02, 0.03]	<0.001	2	[1.49, 2.50]	<0.001	–2.04	[–2.98, –1.10]	<0.001
Age power(NA/0/−5)	-	-	-	1.19	[0.79, 1.58]	<0.001	–0.92	[–1.21, –0.62]	<0.001
ITN use(pregnancy)									
Yes	0	-	-	0	-	-	0	-	-
No	–0.11	[–0.49, 0.26]	0.548	0.16	[–0.22, 0.53]	0.413	0.02	[–0.44, 0.48]	0.918
Season									
Dry season	0	-	-	0	-	-	0	-	-
Rains	0.35	[0.13, 0.57]	0.002	0.33	[0.19, 0.47]	<0.001	0.28	[0.07, 0.50]	0.011
Malaria Exposureindex	0.02	[–0.003, 0.04]	0.098	–0.01	[–0.03, 0.006]	0.202	0.01	[–0.01, 0.04]	0.393
Number previousinfections^b^	0.12	[–0.002, 0.24]	0.053	0.28	[0.17, 0.39]	<0.001	0.31	[0.17, 0.45]	<0.001

a Age is transformed in multiple fractional polynomials with the corresponding powers for antibodies to MSP3, GLURP R0 and GLURP R2 indicated in brackets.

b Number of malaria infections recorded between two consecutive time points for antibody titres measurement.

### Antibody titres and risk of febrile malaria

Anti-MSP3 antibody titres were significantly associated with an increased hazard of the first febrile malaria episode on univariate (Table S4) and multivariable analysis (Table 3). There was no evidence of association between anti-GLURP antibody titres and varying malaria risk. The baseline anti-malaria antibody titres for both antigens did not show any significant association with the risk of febrile malaria. The wet season and individual malaria exposure index were associated with higher risk of febrile malaria. The baseline foetal haemoglobin fraction showed a protective effect in the multivariable model (HR = 0.97, *p* = 0.003, 95% CI [0.96, 0.99]) which was not significant in univariate analysis (Table S3). This was dependent on adjusting for exposure index in the multivariable model, and we noted a non-significant association between foetal haemoglobin and exposure index (r = 0.17, *p* = 0.064). Belonging to the haemoglobin CC type group was also significantly associated with decreased malaria incidence (IRR = 0.44, *p* = 0.046, 95% CI [0.19, 0.99]) in the multivariable but not in the univariate analysis.

**Table 3:** Multivariable models of risk of malaria using changing anti-malaria antibody titres.

	Cox regression			Negative binomial regression		
Predictor^f^	HR^d^	95%CI	*P*	IRR^e^	95%CI	*P*
Age	NA	-	-	1.11	[1.09, 1.13]	<0.001
MUAC^a^ (baseline)	-	-	-	1.26	[1.09, 1.45]	0.002
Foetal Hb^b^ rate (baseline)	0.97	[0.96, 0.99]	0.003	0.98	[0.97, 0.99]	0.013
Haemoglobin type						
AA	1	-	-	1	-	-
AS^c^	NA^c^	-	-	NA^c^	-	-
AC	1.29	[0.68, 2.47]	0.430	1.14	[0.75, 1.72]	0.540
CC	0.52	[0.12, 2.19]	0.370	0.44	[0.19, 0.99]	0.046
Anti-MSP3 (changing)	1.34	[1.08, 1.66]	0.007	1.17	[1.04, 1.30]	0.007
Anti-GLURP R0 (changing)	1.15	[0.91, 1.44]	0.233	1.003	[0.88, 1.14]	0.968
Anti-GLURP R2 (changing)	0.98	[0.83, 1.16]	0.859	0.92	[0.82, 1.03]	0.149
ITN use (pregnancy)						
Yes	1	-	-	1	-	-
No	0.86	[0.41, 1.79]	0.687	1.23	[0.80, 1.88]	0.348
Season						
Dry season	1	-	-	1	-	-
Rains	10.85	[2.80, 42.15]	0.001	1.4	[1.02, 1.92]	0.037
Malaria Exposure index	1.08	[1.04, 1.13]	<0.001	1.06	[1.03, 109]	<0.001

a MUAC: mid upper arm circumference.

b Hb: haemoglobin.

c NA not applicable; only one child had haemoglobin AS type.

d HR: hazard ratio.

e IRR: incidence rate ratio.

f There was no collinearity between the predictors (Figure S5, Table S6).

**Figure 2:**
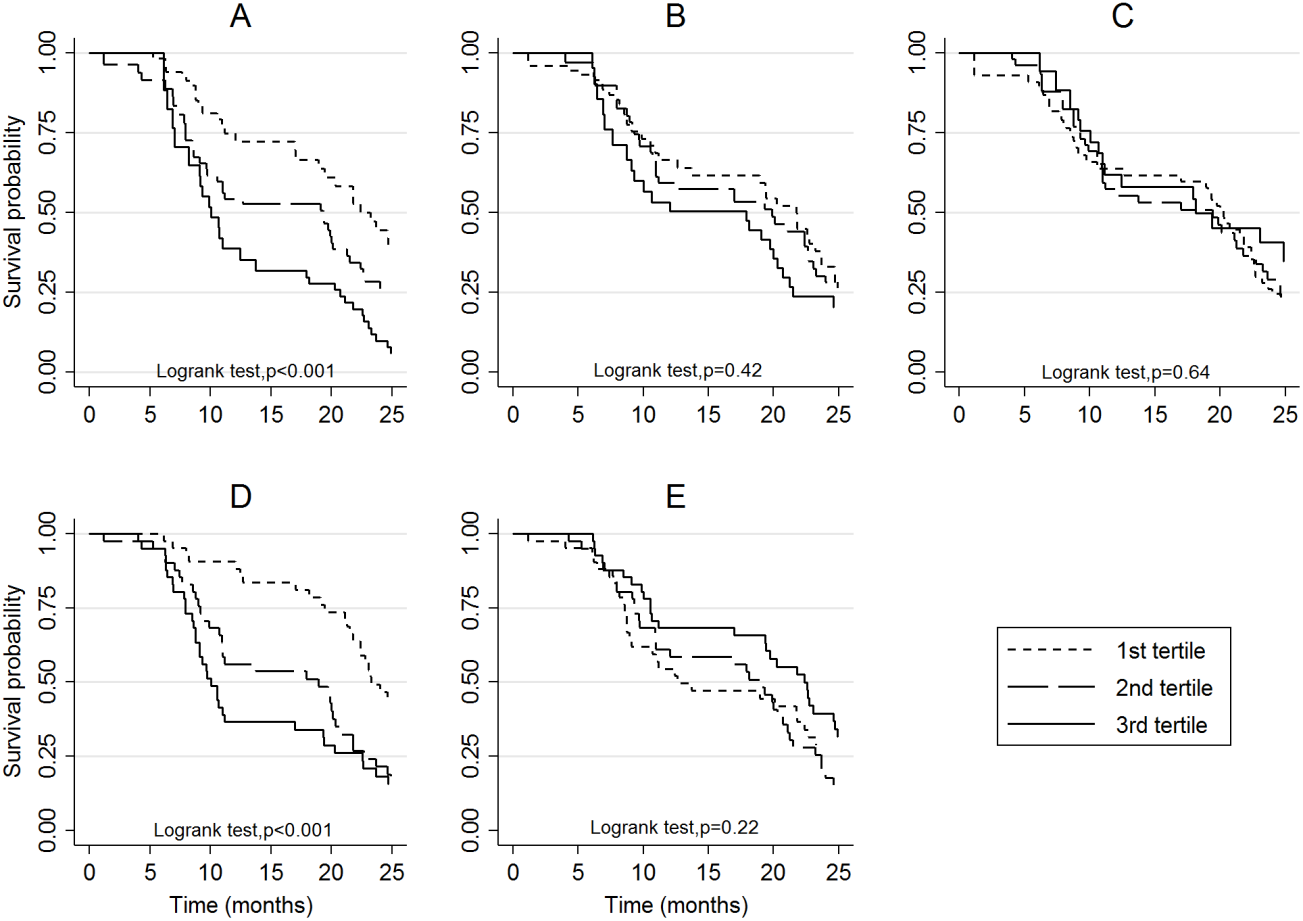
Effect of antibody titres, individual malaria exposure index and baseline foetal haemoglobin rate on survival to malaria: Kaplan Meier estimates. (A) Antibodies to MSP3, (B) Antibodies to GLURP R0, (C) Antibodies to GLURP R2, (D) Individual malaria exposure index, (E) Foetal haemoglobin fraction.

We did not detect significant variation in the proportionality of hazards over time (Figure 2, Figure S4), but there was borderline variation in varying hazards for the exposure index (*p* = 0.082, in the direction of decreasing hazard over time) and for foetal haemoglobin (*p* = 0.098, in the direction of increasing hazard over time).

The results of negative binomial regression were consistent with the Cox regression analysis (Table 3). The best fit lines from the multivariable fractional polynomial regression are consistent with a linear effect of antibody titres on febrile malaria episodes (Figure S6). Changing anti-MSP3 antibody titres were significantly associated with increased incidence of febrile malaria episodes (IRR = 1.17, p = 0.007, 95% CI [1.04, 1.30]), but baseline titres were not. Changing Anti-GLURP R0 antibody titres were significantly associated with increased incidence of febrile malaria on univariate (Table S4) but not multivariable analysis (Table 3). Age, season and individual malaria exposure index were significantly associated with increased incidence of febrile malaria episodes. We examined interaction terms: we found that the effects of total IgG to the malaria antigens we tested were not additive, and noted an interaction between malaria exposure index and total IgG to MSP3 of marginal significance (Table S7).

Restricting the observation period to six weeks post bleeding for plasma, to take into account the fact that anti-malaria antibodies are more likely to be short-lived in young children [50], yielded stronger associations in univariate analysis but not in the multivariable analysis and not in the direction of protection (Table S5).

## Discussion

In this study, transplacentally acquired anti-malaria antibodies and foetal haemoglobin were investigated in relation to susceptibility to malaria in a cohort of 140 infants. Antibody titres to GLURP and MSP3 were found to decline in the first four months of life, presumably due to the loss of maternal antibodies. Endogenous production was responsible for the subsequent increase in the case of GLURP and stabilisation of the loss of antibodies for MSP3. Contrary to our expectations, there was no association between antibody titres to GLURP (R0 and R2) and the risk of febrile malaria in the first two years of life; antibody titres to MSP3 even appeared as a marker of exposure since it was statistically significantly positively associated with the incidence of febrile malaria and inversely associated with time to first febrile malaria episode. Confounding between malaria risk and antibody titre by variation in exposure has previously been reported [51], [52].

In the investigation of the role of antibodies to MSP3 and GLURP (R0 and R2) against *P. falciparum* malaria, most of the previous studies did not specifically target infants. A number of prospective sero-epidemiological studies have investigated the role of antibodies to these merozoite surface antigens in older children and adults in West Africa, East Africa as well as South-East Asia.

The lack of association between antibodies to GLURP (R0 and R2) and protection against clinical malaria in our study is partially concordant with the findings of a previous study in older children in Burkina Faso. Nebie and colleagues investigated total IgG to GLURP (R0 and R2) among others antigens in children aged 6 months to 10 years and found a protective effect for antibodies to GLURP R0 but not to GLURP R2 when the antibody titres were analysed individually. When antibody titres to all the four antigens studied (NANP, GLURP R0 and R2, MSP3) were included in a multivariable model, antibodies to GLURP (R0 and R2) were not associated with protection against malaria [24]. In Ghana, Dodoo and colleagues reported a protective effect of total IgG and IgG subclasses to GLURP (R0 and R2) in the univariate analysis. However neither total IgG nor IgG subclasses to GLURP (R0 and R2) were significantly protective when all the serological covariates were taken into account in the final multivariable model [19], [25]. In a study conducted in Tanzania by Lusingu and colleagues, total IgG to GLURP R0 was not associated with protection against febrile malaria; among IgG subclasses only IgG1 was associated with protection. In contrast, other studies have demonstrated a protective association for antibodies to GLURP (R0 and R2) in older children and adults [20], [26], [27], [30]. In a study in Myanmar that investigated antibodies to MSP1, MSP3, GLURP (R0, R1, R2), only antibodies to GLURP R0 showed a protective effect when all the antibodies were considered together in the analysis [21].

Antibodies to MSP3 have been associated with protection in previous sero-epidemiological studies [21], [23], [27], [53] and a vaccine trial [44] even if the assessment of efficacy was not the primary objective in the latter. However other studies did not find a protective effect of antibodies to MSP3 [19], [25], [26] but none of these studies concluded on antibodies to MSP3 appearing as a marker of exposure.

Although antibodies to MSP3 and GLURP have not been previously studied in newborn cohorts, antibodies to other *P. falciparum* malaria antigens have been investigated. In sero-epidemiological newborn cohort studies, antibodies to crude *P. falciparum* schizont extract and MSP2 were found to be associated with higher risk of malaria infection in infants [38], [40] indicating higher exposure. Only antibodies to MSP1-19 were associated with protection against clinical malaria [42], [43].

Antibody titres to MSP3 and GLURP (R0 and R2) were not associated with protection in our study, and we suggest that confounding due to exposure led to an apparent association with increased susceptibility for antibody response to MSP3. Limitations of our study include the fact that the high malaria transmission season began 5–7 months after recruitment. Therefore the majority of the maternal antibodies were likely gone by the time that febrile malaria episodes began, and children were exposed during a period of lower antibody titres. We did not use an external control to quantify malaria antibodies as performed elsewhere [54], however we speculate that antibody titres at 5 months and beyond were lower than those previously reported to be protective [42], [43].

Interestingly, foetal haemoglobin was significantly inversely associated to febrile malaria incidence although the effect size was relatively small. The effect was only statistically significant on multivariable analysis, and appeared to depend on adjusting by exposure index in the multivariable model. Furthermore the effect seems to be evident after 6 months of age, when we would expect foetal haemoglobin to have been lost from the circulation. We speculate that an interaction between malaria exposure and foetal haemoglobin may be responsible for a delayed protective effect, perhaps due to an early but controlled infection in the presence of high levels of foetal haemoglobin leading to more rapid acquisition of immunity [55].

Children who carried the haemoglobin CC type appeared to have a significantly lower risk of malaria as compared to haemoglobin AA type children, as has been previously reported [56], [57].

In conclusion, the present study did not find any evidence of association between antibody titres to MSP3 and GLURP (R0 and R2) and protection against *P. falciparum* febrile malaria in children in their first few months of life. Despite the above mentioned limitations of the study, the baseline fraction of foetal was associated with protection against febrile malaria. Its role in the protection of children against malaria in their first few months of life is therefore worthy of further investigation. Finally, our study also underlines that the role of haemoglobinopathies should be taken into account in the exploration of protective factors in the low susceptibility of infants to malaria.

## Acknowledgments

We are grateful to the parents/guardians of the children in Banfora whose commitment and patience made this study possible. We thank the fieldworkers’ team and nurses in the dispensaries who monitored these children for two years as well as all the lab staff. We would also like to especially thank Dr Michael Theisen who kindly provided the malaria antigens used in this study, MSP3-LSP, GLURP R0 and GLURP R2.

## Supporting Information

Figure S1 **Distribution of parasitemia in febrile and afebrile children for the whole study period.** The box-and-whisker plot represents the median and the inter-quartile range of the parasite density in a log 10 scale in the febrile and afebrile children groups.
(TIF)

Figure S2 **Selection of the best radius for individual malaria exposure index calculation.** The lowest log likelihood in the univariate Cox regression analysis was the selection criteria for the radius to be used.
(TIF)

Figure S3 **Dynamics of total IgG to MSP3 over the whole study period using a 25% random sample representing 23 children.** Each line represents a child. The antibody titres are in log 10 scale.
(TIF)

Figure S4 **Test of proportional hazards assumption: Schoenfeld residuals plots.** (A) Baseline total IgG to MSP3, (B) Baseline total IgG to GLURP R0, (C) Baseline total IgG to GLURP R2, (D) Changing total IgG to MSP3, (E) Changing total IgG to GLURP R0, (F) Changing total IgG to GLURP R2, (G) Individual malaria exposure index, (H) Baseline Foetal haemoglobin rate.
(TIF)

Figure S5 **Scatter plot matrix of the continuous independent variables used used in multivariable regression models.**
(TIF)

Figure S6 **Multivariable fractional polynomial plots for antibodies to MSP3 (A), GLURP R0 (B) and GLURP R2 (C).**
(TIF)

Table S1 **Variability of antibody titres dynamics.** The correlations (r) are examined between every two consecutive time points for antibody measurement.
(DOC)

Table S2 **Predictive model for changing anti-malaria antibody titers using linear regression. Univariate analysis.**
(DOCX)

Table S3 **Cox regression analysis using changing antibody titres.**
(DOCX)

Table S4 **Predictive model for the occurrence of febrile malaria episodes using changing antibody** **titres.**
(DOCX)

Table S5 **Predictive model for the occurrence of febrile malaria episodes using changing antibody titres with a restricted observation period (6 weeks) post-bleeding for plasma.**
(DOCX)

Table S6 **Multicollinearity diagnostics for continuous independent variables used in multivariable regression models.**
(DOCX)

Table S7 **Testing for interactions between predictors in multivariable negative binomial regression model.**
(DOCX)
