# Supplementary figures and images for: Plasmodium falciparum Malaria in Children Aged 0-2 Years: The Role of Foetal Haemoglobin and Maternal Antibodies to Two Asexual Malaria Vaccine Candidates (MSP3 and GLURP)
Source: PLoS One. 2014 Sep 19;9(9):e107965. doi: 10.1371/journal.pone.0107965 (PMC4169582; doi:10.1371/journal.pone.0107965)

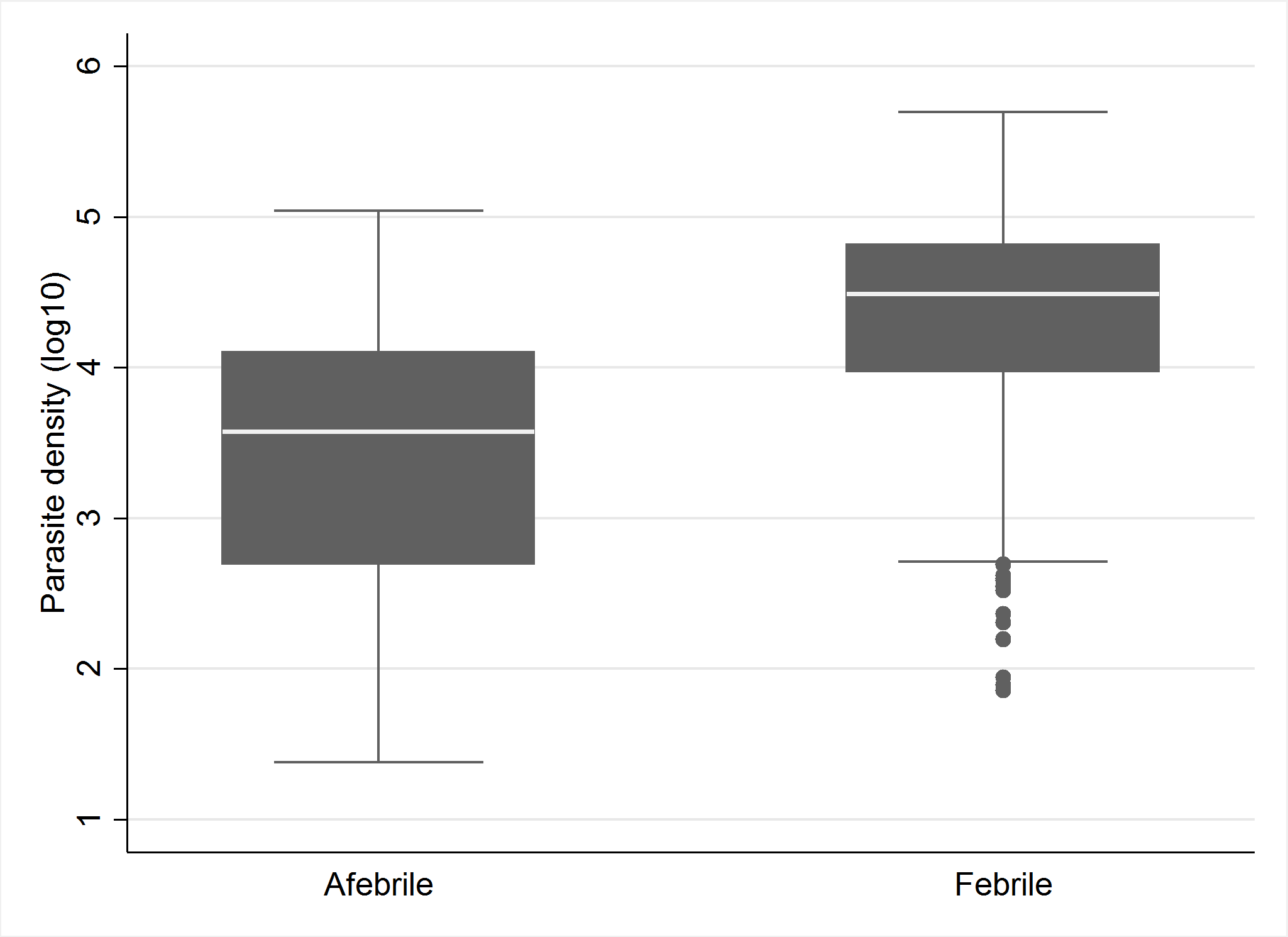

Supplement: Figure S1 — Distribution of parasitemia in febrile and afebrile children for the whole study period. The box-and-whisker plot represents the median and the inter-quartile range of the parasite density in a log 10 scale in the febrile and afebrile children groups. (TIF) [file pone.0107965.s001.tif]

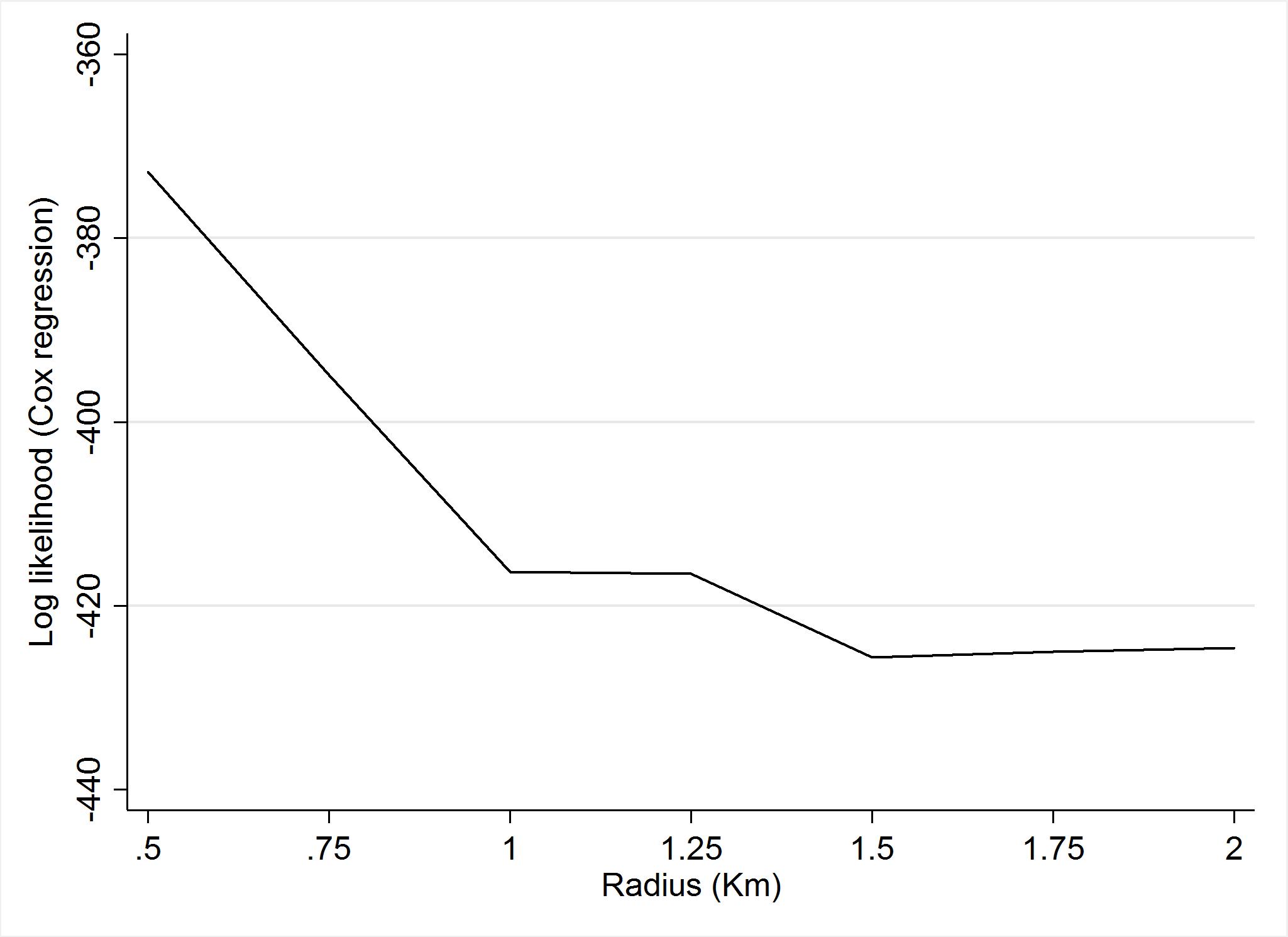

Supplement: Figure S2 — Selection of the best radius for individual malaria exposure index calculation. The lowest log likelihood in the univariate Cox regression analysis was the selection criteria for the radius to be used. (TIF) [file pone.0107965.s002.tif]

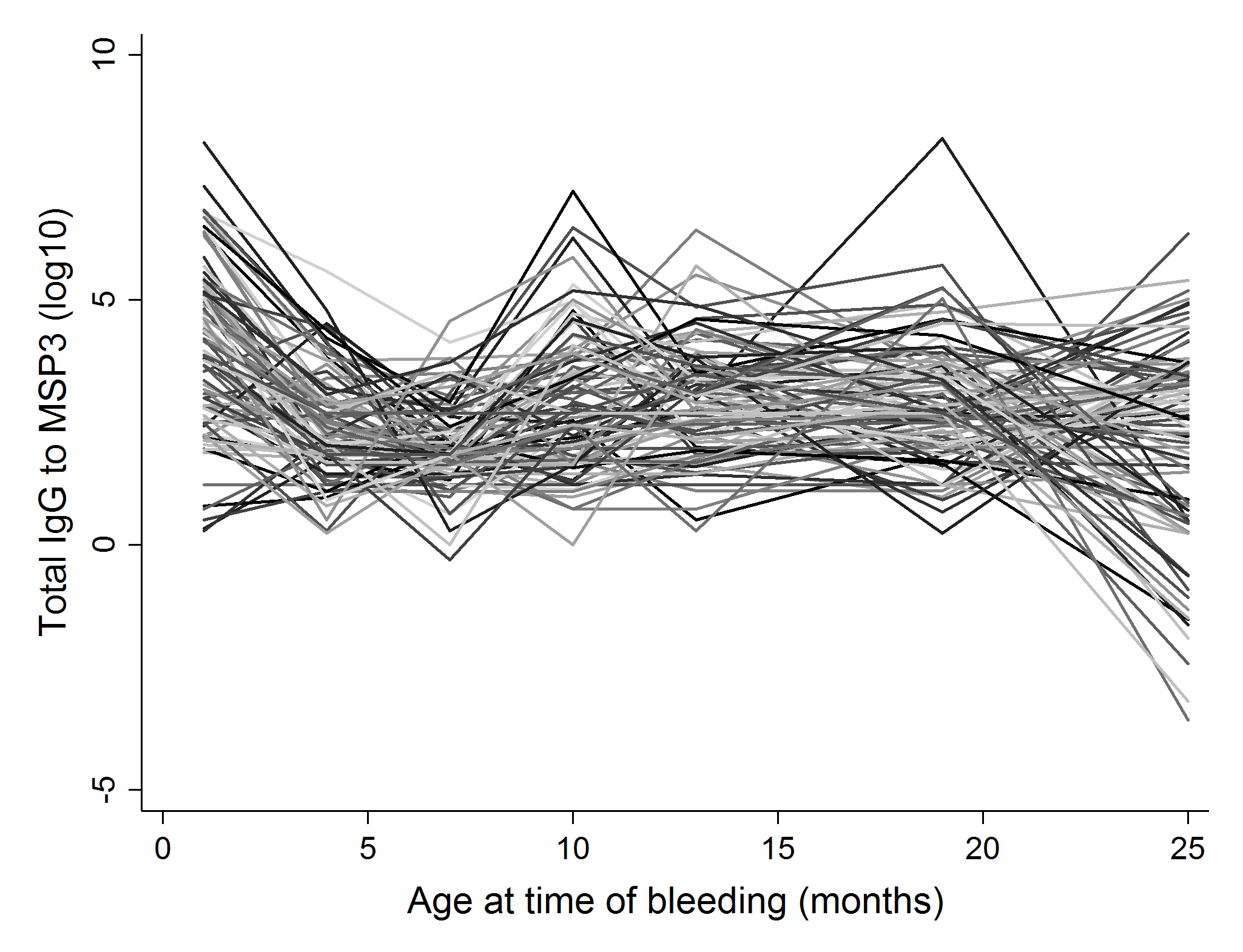

Supplement: Figure S3 — Dynamics of total IgG to MSP3 over the whole study period using a 25% random sample representing 23 children. Each line represents a child. The antibody titres are in log 10 scale. (TIF) [file pone.0107965.s003.tif]

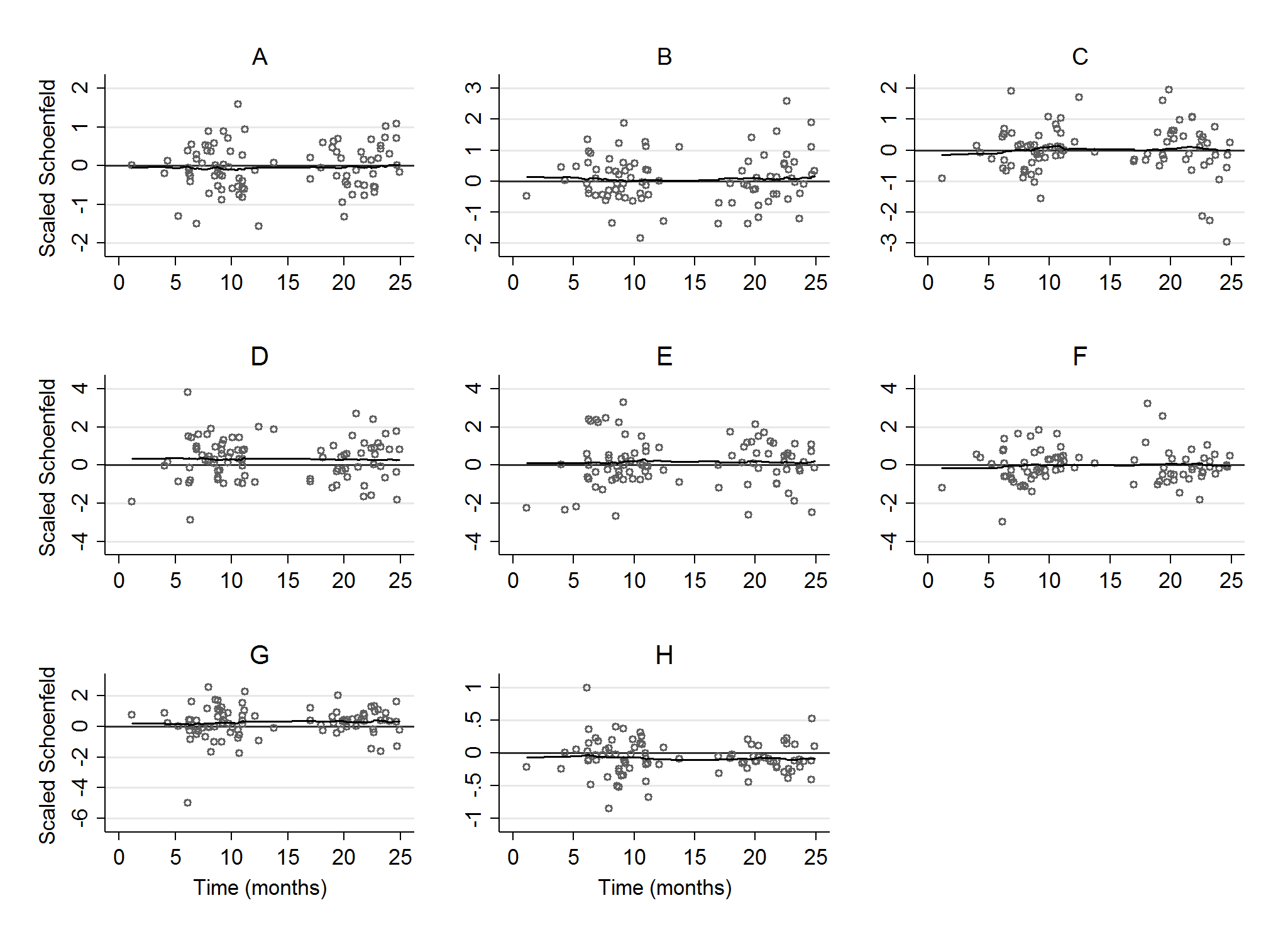

Supplement: Figure S4 — Test of proportional hazards assumption: Schoenfeld residuals plots. (A) Baseline total IgG to MSP3, (B) Baseline total IgG to GLURP R0, (C) Baseline total IgG to GLURP R2, (D) Changing total IgG to MSP3, (E) Changing total IgG to GLURP R0, (F) Changing total IgG to GLURP R2, (G) Individual malaria exposure index, (H) Baseline Foetal haemoglobin rate. (TIF) [file pone.0107965.s004.tif]

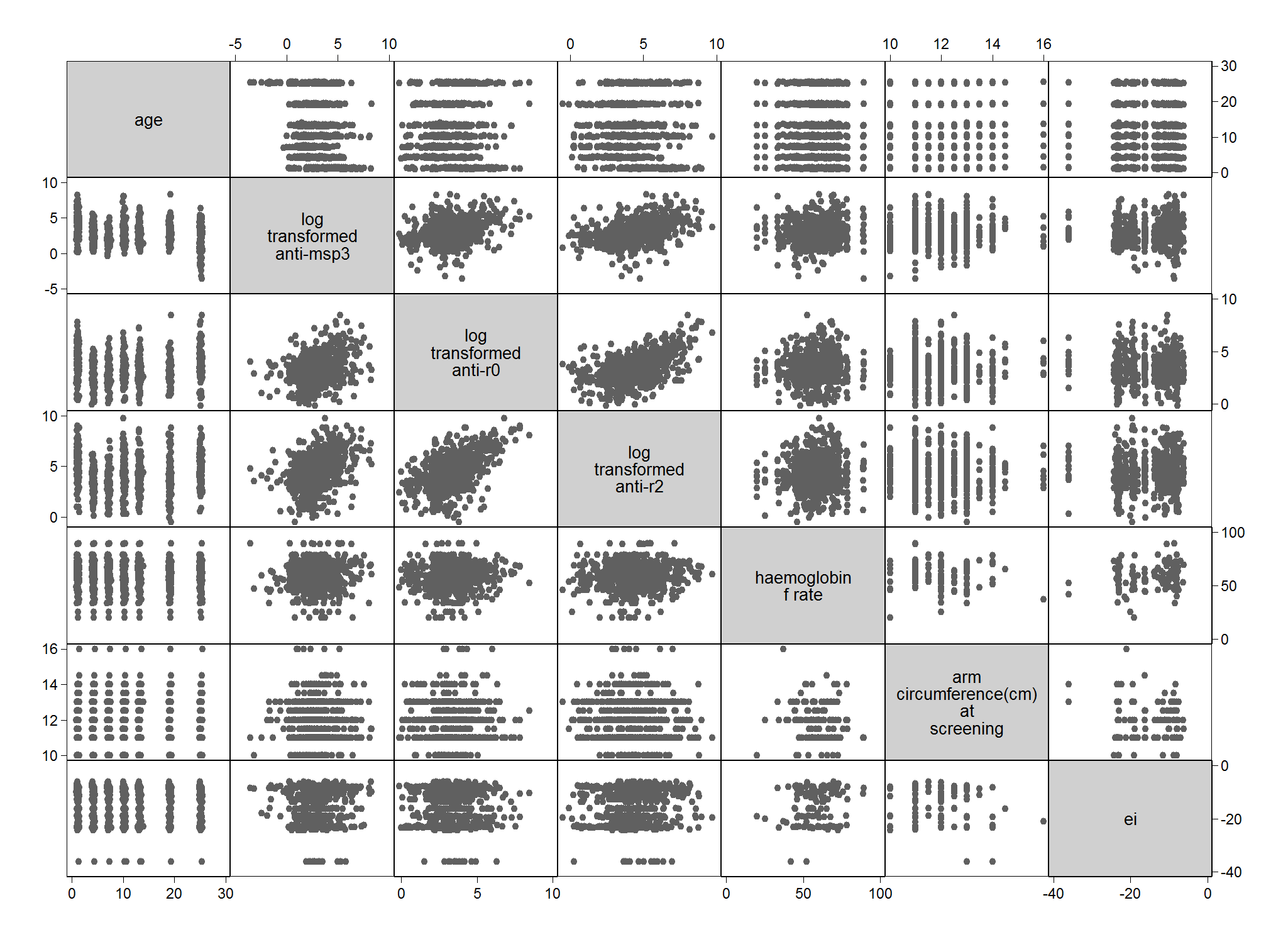

Supplement: Figure S5 — Scatter plot matrix of the continuous independent variables used used in multivariable regression models. (TIF) [file pone.0107965.s005.tif]

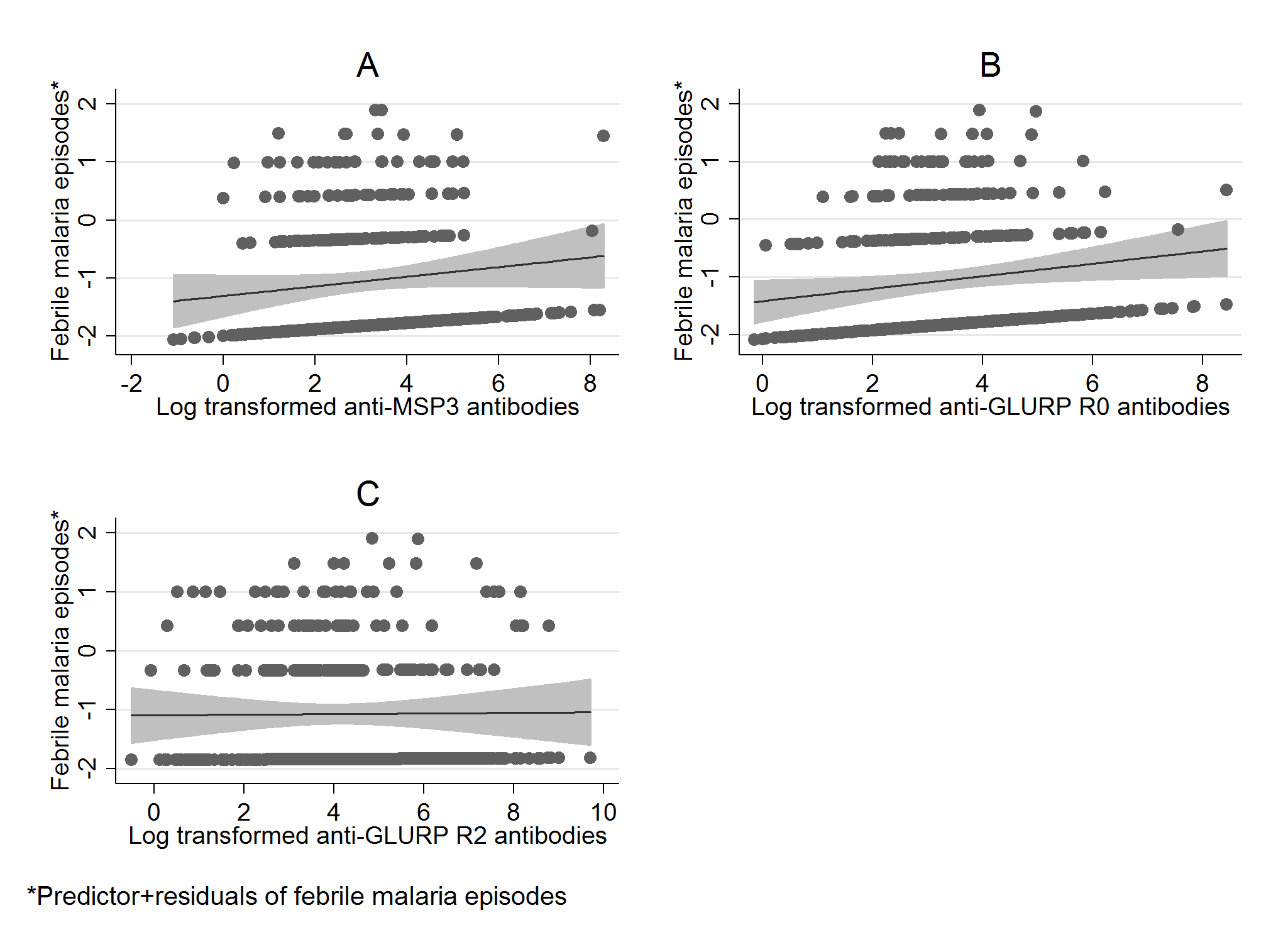

Supplement: Figure S6 — Multivariable fractional polynomial plots for antibodies to MSP3 (A), GLURP R0 (B) and GLURP R2 (C). (TIF) [file pone.0107965.s006.tif]
