# Supplementary material for: Plasmodium falciparum Malaria in Children Aged 0-2 Years: The Role of Foetal Haemoglobin and Maternal Antibodies to Two Asexual Malaria Vaccine Candidates (MSP3 and GLURP)
Source: PLoS One. 2014 Sep 19;9(9):e107965. doi: 10.1371/journal.pone.0107965 (PMC4169582; doi:10.1371/journal.pone.0107965)
Supplement: Table S1 — Variability of antibody titres dynamics. The correlations (r) are examined between every two consecutive time points for antibody measurement. (DOC) [file pone.0107965.s007.doc]

**Table S1**. Variability of antibody titres dynamics. The correlations (r) are examined between every two consecutive time points for antibody measurement.

| Time points (month) | Anti-MSP3* | | Anti-GLURP R0* | | Anti-GLURP R2* | |
| --- | --- | --- | --- | --- | --- | --- |
|  | R | *p* | R | *p* | r | *p* |
| M0🡪M3 | 0.5460 | <0.0001 | 0.5514 | <0.0001 | 0.6022 | <0.0001 |
| M3🡪M6 | 0.1805 | 0.0852 | 0.3410 | 0.0009 | 0.3480 | 0.0007 |
| M6🡪M9 | 0.4015 | 0.0001 | 0.4074 | 0.0001 | 0.2448 | 0.0187 |
| M9🡪M12 | 0.4265 | <0.0001 | 0.5885 | <0.0001 | 0.4444 | <0.0001 |
| M12🡪M18 | 0.3054 | 0.0031 | 0.3657 | 0.0003 | 0.2819 | 0.0065 |
| M18🡪M24 | -0.2800 | 0.0069 | 0.3148 | 0.0022 | 0.1489 | 0.1565 |

*Antibody titres in log2 scale.
