# Supplementary material for: Plasmodium falciparum Malaria in Children Aged 0-2 Years: The Role of Foetal Haemoglobin and Maternal Antibodies to Two Asexual Malaria Vaccine Candidates (MSP3 and GLURP)
Source: PLoS One. 2014 Sep 19;9(9):e107965. doi: 10.1371/journal.pone.0107965 (PMC4169582; doi:10.1371/journal.pone.0107965)
Supplement: Table S2 — Predictive model for changing anti-malaria antibody titers using linear regression. Univariate analysis. (DOCX) [file pone.0107965.s008.docx]

**Table S2**. Predictive model for changing anti-malaria antibody titers using linear regression. Univariate analysis.

|  | **IgG anti-MSP3** | | | **IgG anti-GLURP R0** | | | **IgG anti-GLURP R2** | | |
| --- | --- | --- | --- | --- | --- | --- | --- | --- | --- |
| **Predictor** | **Coef.** | **95% CI** | **p** | **Coef.** | **95%CI** | **p** | **Coef.** | **95%CI** | **p** |
| Age power (-2/-5/-5)* | 0.02 | [0.02, 0.03] | <0.001 | 0.24 | [0.10, 0.39] | 0.001 | 0.51 | [0.36, 0.66] | <0.001 |
| Age power (NA/0/-5)* | - | - | - | -0.10 | [-0.21, 0.02] | 0.093 | -0.19 | [-0.24, -0.14] | <0.001 |
| Sex |  |  |  |  |  |  |  |  |  |
| Male | 0 | - | - | 0 | - | - | 0 | - | - |
| Female | -0.18 | [-0.42, 0.07] | 0.154 | 0.14 | [-0.11, 0.40] | 0.273 | -0.22 | [-0.54, 0.10] | 0.179 |
| Weight (baseline) | -0.04 | [-0.25, 0.17] | 0.723 | -0.06 | [-0.26, 0.15] | 0.589 | -0.20 | [-0.46, 0.07] | 0.139 |
| Length (baseline) | 0.02 | [-0.02, 0.06] | 0.310 | -0.002 | [-0.05, 0.05] | 0.944 | -0.04 | [-0.11, 0.02] | 0.182 |
| MUAC (baseline) | 0.05 | [-0.07, 0.18] | 0.425 | 0.002 | [-0.11, 0.11] | 0.976 | -0.04 | [-0.17, 0.09] | 0.563 |
| Foetal Hb fraction (baseline) | 0.006 | [-0.006, 0.02] | 0.317 | 0.001 | [-0.01, 0.01] | 0.893 | 0.01 | [-0.003, 0.02] | 0.144 |
| Hemoglobin type |  |  |  |  |  |  |  |  |  |
| AA | 0 | - | - | 0 | - | - | 0 | - | - |
| AS** | NA | - | - | NA | - | - | NA | - | - |
| AC | -0.22 | [-0.44, -0.17] | 0.276 | 0.05 | [-0.38, 0.48] | 0.832 | 0.04 | [-0.49, 0.56] | 0.885 |
| CC | 0.04 | [-0.65, 0.73] | 0.911 | 0.01 | [-0.55, 0.58] | 0.963 | 0.36 | [0.003, 0.71] | 0.048 |
| Month of birth |  |  |  |  |  |  |  |  |  |
| October | 0 | - | - | 0 | - | - | 0 | - | - |
| November | 0.30 | [-0.07, 0.66] | 0.108 | 0.17 | [-0.25, 0.59] | 0.421 | 0.07 | [-0.40, 0.55] | 0.757 |
| December | 0.07 | [-0.30, 0.44] | 0.724 | 0.10 | [-0.32, 0.52] | 0.636 | -0.30 | [-0.80, 0.20] | 0.235 |
| January | -0.02 | [-0.41, 0.38] | 0.927 | 0.30 | [-0.19, 0.80] | 0.222 | 0.14 | [-0.40, 0.68] | 0.603 |
| EPI status (baseline) |  |  |  |  |  |  |  |  |  |
| Up to date | 0 | - | - | 0 | - | - | 0 | - | - |
| Not up to date | 0.22 | [-0.20, 0.63] | 0.307 | 0.29 | [-0.08, 0.66] | 0.121 | -0.01 | [-0.45, 0.43] | 0.961 |
| Age mother (baseline) | 0.02 | [-0.001, 0.04] | 0.057 | 0.01 | [-0.005, 0.03] | 0.138 | 0.02 | [-0.01, 0.04] | 0.166 |
| Gravidity status |  |  |  |  |  |  |  |  |  |
| Primigravidae | 0 | - | - | 0 | - | - | 0 | - | - |
| Multigravidae | 0.18 | [-0.11, 0.47] | 0.213 | 0.05 | [-0.20, 0.29] | 0.704 | 0.01 | [-0.34, 0.37] | 0.945 |
| ITN use (pregnancy) |  |  |  |  |  |  |  |  |  |
| Yes | 0 | - | - | 0 | - | - | 0 | - | - |
| No | -0.10 | [-0.47, 0.27] | 0.599 | 0.15 | [-0.26, 0.55] | 0.471 | 0.21 | [-0.26, 0.68] | 0.372 |
| IPTp courses |  |  |  |  |  |  |  |  |  |
| 0 | 0 | - | - | 0 | - | - | 0 | - | - |
| 1 | 0.56 | [0.17, 0.94] | 0.005 | -0.22 | [-0.93, 0.50] | 0.549 | -0.73 | [-1.49, 0.03] | 0.060 |
| 2 | 0.35 | [0.07, 0.64] | 0.017 | -0.24 | [-0.89, 0.40] | 0.462 | -0.75 | [-1.47, -0.03] | 0.040 |
| 3 | 0.20 | [-0.06, 0.45] | 0.127 | -0.12 | [-0.81, 0.57] | 0.737 | -1.88 | [-3.06, -0.70] | 0.002 |
| Education level (mother) |  |  |  |  |  |  |  |  |  |
| None | 0 | - | - | 0 | - | - | 0 | - | - |
| Primary | 0.18 | [-0.07, 0.43] | 0.150 | -0.07 | [-0.38, 0.25] | 0.648 | -0.21 | [-0.57, 0.16] | 0.260 |
| Secondary or above | -0.36 | [-0.76, 0.08] | 0.116 | -0.31 | [-0.63, 0.01] | 0.057 | -0.50 | [-0.87, -0.12] | 0.010 |
| Zone of residence |  |  |  |  |  |  |  |  |  |
| Rural | 0 | - | - | 0 | - | - | 0 | - | - |
| Urban | -0.25 | [-0.53, 0.02] | 0.074 | -0.44 | [-0.72, -0.15] | 0.003 | -0.70 | [-1.05, -0.36] | <0.001 |
| Mixed | 0.21 | [-0.10, 0.53] | 0.175 | -0.43 | [-0.77, -0.10] | 0.010 | -0.35 | [-0.74, 0.03] | 0.072 |
| Season |  |  |  |  |  |  |  |  |  |
| Dry season | 0 | - | - | 0 | - | - | 0 | - | - |
| Rains | -0.10 | [-0.32, 0.12] | 0.355 | 0.11 | [-0.04, 0.26] | 0.133 | -0.08 | [-0.31, 0.14] | 0.461 |
| Malaria Exposure index | 0.03 | [0.01, 0.05] | 0.011 | -0.0003 | [-0.02, 0.02] | 0.976 | 0.02 | [-0.002, 0.05] | 0.069 |
| Number previous infections | 0.05 | [-0.06, 0.17] | 0.373 | 0.28 | [0.17, 0.38] | <0.001 | 0.28 | [0.14, 0.41] | <0.001 |

*Powers of multiple fractional polynomials of age

**Only one participant had haemoglobin phenotype AS
