# Supplementary material for: Plasmodium falciparum Malaria in Children Aged 0-2 Years: The Role of Foetal Haemoglobin and Maternal Antibodies to Two Asexual Malaria Vaccine Candidates (MSP3 and GLURP)
Source: PLoS One. 2014 Sep 19;9(9):e107965. doi: 10.1371/journal.pone.0107965 (PMC4169582; doi:10.1371/journal.pone.0107965)
Supplement: Table S3 — Cox regression analysis using changing antibody titres. (DOCX) [file pone.0107965.s009.docx]

**Table S3**. Cox regression analysis using changing antibody titres.

|  | **Univariate analysis** | | | **Multivariable analysis** | | |
| --- | --- | --- | --- | --- | --- | --- |
| **Predictor** | **HR** | **95% CI** | **p** | **HR** | **95%CI** | **p** |
| Sex |  |  |  |  |  |  |
| Male | 1 | - | - | - | - | - |
| Female | 0.86 | [0.57, 1.30] | 0475 | - | - | - |
| Weight (baseline) | 1.06 | [0.75, 1.50] | 0.754 | - | - | - |
| Length (baseline) | 1.03 | [0.95, 1.13] | 0.442 | - | - | - |
| MUAC (baseline) | 1.13 | [0.95, 1.34] | 0.182 | - | - | - |
| Foetal Hb fraction (baseline) | 0.98 | [0.97, 1.001] | 0.075 | 0.97 | [0.96, 0.99] | 0.003 |
| Hemoglobin type |  |  |  |  |  |  |
| AA | 1 | - | - | 1 | - | - |
| AS* | NA | - | - | NA | - | - |
| AC | 1.30 | [0.71, 2.39] | 0.401 | 1.29 | [0.68, 2.47] | 0.43 |
| CC | 0.50 | [0.12, 2.03] | 0.330 | 0.52 | [0.12, 2.19] | 0.37 |
| Anti-MSP3 (changing) | 1.41 | [1.16, 1.70] | <0.001 | 1.34 | [1.08, 1.66] | 0.007 |
| Anti-GLURP R0 (changing) | 1.11 | [0.90, 1.37] | 0.326 | 1.15 | [0.91, 1.44] | 0.233 |
| Anti-GLURP R2 (changing) | 1.03 | [0.89, 1.20] | 0.664 | 0.98 | [0.83, 1.16] | 0.859 |
| Anti-MSP3 (baseline) | 0.96 | [0.85, 1.07] | 0.460 |  |  |  |
| Anti-GLURP R0 (baseline) | 1.01 | [0.87, 1.16] | 0.945 |  |  |  |
| Anti-GLURP R2 (baseline) | 1.04 | [0.92, 1.18] | 0.534 |  |  |  |
| Month of birth |  |  |  |  |  |  |
| October | 1 | - | - | - | - | - |
| November | 1.41 | [0.64, 3.08] | 0.392 | - | - | - |
| December | 1.33 | [0.61, 2.88] | 0.476 | - | - | - |
| January | 2.49 | [1.10, 5.64] | 0.029 | - | - | - |
| EPI status (baseline) |  |  |  |  |  |  |
| Up to date | 1 | - | - | - | - | - |
| Not up to date | 1.24 | [0.69, 2.23] | 0.476 | - | - | - |
| Age mother (baseline) | 1.02 | [0.98, 1.05] | 0.348 | - | - | - |
| Gravidity status |  |  |  |  |  |  |
| Primigravidae | 1 | - | - | - | - | - |
| Multigravidae | 0.99 | [0.59, 1.64] | 0.957 | - | - | - |
| ITN use (pregnancy) |  |  |  |  |  |  |
| Yes | 1 | - | - | 1 | - | - |
| No | 0.87 | [0.44, 1.74] | 0.703 | 0.86 | [0.41, 1.79] | 0.687 |
| IPTp courses |  |  |  |  |  |  |
| 0 | 1 | - | - | - | - | - |
| 1 | 1.71 | [0.62, 4.53] | 0.284 | - | - | - |
| 2 | 1.26 | [0.51, 3.14] | 0.618 | - | - | - |
| 3 | 0.65 | [0.08, 5.54 ] | 0.691 | - | - | - |
| Education level (mother) |  |  |  |  |  |  |
| None | 1 | - | - | - | - | - |
| Primary | 1.39 | [0.88, 2.17] | 0.156 | - | - | - |
| Secondary or above | 0.64 | [0.33, 1.27] | 0.203 | - | - | - |
| Zone of residence |  |  |  |  |  |  |
| Rural | 1 | - | - | - | - | - |
| Urban | 0.43 | [0.25, 0.73] | 0.002 | - | - | - |
| Mixed | 1.32 | [0.82, 2.14] | 0.252 | - | - | - |
| Season |  |  |  |  |  |  |
| Dry season | 1 |  |  | 1 |  |  |
| Rains | 10.10 | [2.83, 36] | <0.001 | 10.85 | [2.80, 42.15] | 0.001 |
| Malaria Exposure index | 1.08 | [1.04, 1.11] | <0.001 | 1.08 | [1.04, 1.13] | <0.001 |

*Only one participant had haemoglobin phenotype AS
