# Supplementary material for: Plasmodium falciparum Malaria in Children Aged 0-2 Years: The Role of Foetal Haemoglobin and Maternal Antibodies to Two Asexual Malaria Vaccine Candidates (MSP3 and GLURP)
Source: PLoS One. 2014 Sep 19;9(9):e107965. doi: 10.1371/journal.pone.0107965 (PMC4169582; doi:10.1371/journal.pone.0107965)
Supplement: Table S4 — Predictive model for the occurrence of febrile malaria episodes using changing antibody titres. (DOCX) [file pone.0107965.s010.docx]

**Table S4**. Predictive model for the occurrence of febrile malaria episodes using changing antibody titres.

|  | **Univariate analysis** | | | **Multivariable analysis** | | |
| --- | --- | --- | --- | --- | --- | --- |
| **Predictor** | **IRR** | **95% CI** | **p** | **IRR** | **95%CI** | **p** |
| Age | 1.12 | [1.10, 1.13] | <0.001 | 1.11 | [1.09, 1.13] | <0.001 |
| Sex |  |  |  |  |  |  |
| Male | 1 | - | - | - | - | - |
| Female | 0.87 | [0.63, 1.21] | 0.418 | - | - | - |
| Weight (baseline) | 1.10 | [0.83, 1.44] | 0.509 | - | - | - |
| Length (baseline) | 1.03 | [0.98, 1.10] | 0.259 | - | - | - |
| MUAC (baseline) | 1.22 | [1.04, 1.42] | 0.012 | 1.26 | [1.09, 1.45] | 0.002 |
| Foetal Hb fraction (baseline) | 0.99 | [0.98, 1.003] | 0.133 | 0.98 | [0.97, 0.99] | 0.013 |
| Hemoglobin type |  |  |  |  |  |  |
| AA | 1 | - | - | 1 | - | - |
| AS* | NA | - | - | NA | - | - |
| AC | 1.15 | [0.76,1.74] | 0.510 | 1.14 | [0.75, 1.72] | 0.540 |
| CC | 0.65 | [1.16, 2.69] | 0.557 | 0.44 | [0.19, 0.99] | 0.046 |
| Anti-MSP3 (changing) | 1.12 | [1.01, 1.24] | 0.037 | 1.17 | [1.04, 1.30] | 0.007 |
| Anti-GLURP R0 (changing) | 1.12 | [1.02, 1.24] | 0.021 | 1.003 | [0.88, 1.14] | 0.968 |
| Anti-GLURP R2 (changing) | 1.01 | [0.92, 1.10] | 0.915 | 0.92 | [0.82, 1.03] | 0.149 |
| Anti-MSP3 (baseline) | 0.99 | [0.91, 1.10] | 0.986 | - | - | - |
| Anti-GLURP R0 (baseline) | 1.05 | [0.95, 1.17] | 0.331 | - | - | - |
| Anti-GLURP R2 (baseline) | 1.08 | [0.99, 1.18] | 0.090 | - | - | - |
| Month of birth |  |  |  |  |  |  |
| October | 1 | - | - | 1 | - | - |
| November | 0.44 | [0.22, 0.88] | 0.020 | - | - |  |
| December | 0.61 | [0.40, 0.92] | 0.018 | - | - | - |
| January | 0.66 | [0.44, 0.98] | 0.040 | - | - | - |
| EPI status (baseline) |  |  |  |  |  |  |
| Up to date | 1 | - | - | 1 | - | - |
| Not up to date | 1.38 | [0.95, 1.99] | 0.089 | - | - | - |
| Age mother (baseline) | 1.02 | [1, 1.05] | 0.055 | - | - | - |
| Gravidity status |  |  |  |  |  |  |
| Primigravidae | 1 | - | - | 1 | - | - |
| Multigravidae | 1.16 | [0.78, 1.74] | 0.470 | - | - | - |
| ITN use (pregnancy) |  |  |  |  |  |  |
| Yes | 1 | - | - | 1 | - | - |
| No | 1.11 | [0.65, 1.91] | 0.705 | 1.23 | [0.80, 1.88] | 0.348 |
| IPTp courses |  |  |  |  |  |  |
| 0 | 1 | - | - | 1 | - | - |
| 1 | 2.04 | [0.89, 4.67] | 0.090 | - | - | - |
| 2 | 1.5 | [0.68, 3.31] | 0.314 | - | - | - |
| 3 | 0.71 | [0.13, 3.79] | 0.684 | - | - | - |
| Education level (mother) |  |  |  |  |  |  |
| None | 1 | - | - | 1 | - | - |
| Primary | 1.21 | [0.87, 1.68] | 0.254 | 1.16 | [0.85, 1.59] | 0.355 |
| Secondary or above | 0.33 | [0.20, 0.55] | <0.001 | 0.47 | [0.26, 0.87] | 0.016 |
| Zone of residence |  |  |  |  |  |  |
| Rural | 1 | - | - | 1 | - | - |
| Urban | 0.38 | [0.24, 0.59] | <0.001 | - | - | - |
| Mixed | 1.15 | [0.84, 1.58] | 0.383 | - | - | - |
| Season |  |  |  |  |  |  |
| Dry season | 1 | - | - | 1 | - | - |
| Rains | 2.02 | [1.48, 2.76] | <0.001 | 1.4 | [1.02, 1.92] | 0.037 |
| Malaria Exposure index | 1.06 | [1.03, 1.10] | <0.001 | 1.06 | [1.03, 1.09] | <0.001 |

*Only one participant had haemoglobin phenotype AS
