# Supplementary material for: Plasmodium falciparum Malaria in Children Aged 0-2 Years: The Role of Foetal Haemoglobin and Maternal Antibodies to Two Asexual Malaria Vaccine Candidates (MSP3 and GLURP)
Source: PLoS One. 2014 Sep 19;9(9):e107965. doi: 10.1371/journal.pone.0107965 (PMC4169582; doi:10.1371/journal.pone.0107965)
Supplement: Table S5 — Predictive model for the occurrence of febrile malaria episodes using changing antibody titres with a restricted observation period (6 weeks) post-bleeding for plasma. (DOCX) [file pone.0107965.s011.docx]

**Table S5**. Predictive model for the occurrence of febrile malaria episodes using changing antibody titres with a restricted observation period (6 weeks) post-bleeding for plasma.

|  | **Univariate analysis** | | | **Multivariable analysis** | | |
| --- | --- | --- | --- | --- | --- | --- |
| **Predictor** | **IRR** | **95% CI** | **p** | **IRR** | **95%CI** | **p** |
| Age | 1.09 | [1.06, 1.12] | <0.001 | 1.06 | [1.03, 1.10] | <0.001 |
| Sex |  |  |  |  |  |  |
| Male | 1 | - | - | - | - | - |
| Female | 0.72 | [0.46, 1.13] | 0.150 | - | - | - |
| Weight (baseline) | 1.24 | [0.88, 1.76] | 0.221 | - | - | - |
| Length (baseline) | 1.06 | [0.97, 1.17] | 0.195 | - | - | - |
| MUAC (baseline) | 1.31 | [1.11, 1.54] | 0.001 | 1.37 | [1.17, 1.60] | <0.001 |
| Foetal Hb fraction (baseline) | 0.99 | [0.98, 1.01] | 0.818 | 0.99 | [0.98, 1.01] | 0.520 |
| Hemoglobin type |  |  |  |  |  |  |
| AA | 1 | - | - | 1 | - | - |
| AS* | NA | - | - | NA | - | - |
| AC | 1.58 | [0.97,2.58] | 0.065 | 1.57 | [0.96, 2.57] | 0.073 |
| CC | 0.30 | [0.05, 1.67] | 0.169 | 0.28 | [0.06, 1.36] | 0.118 |
| Anti-MSP3 (changing) | 1.18 | [1.07, 1.29] | 0.001 | 1.08 | [0.95, 1.23] | 0.258 |
| Anti-GLURP R0 (changing) | 1.37 | [1.23, 1.53] | <0.001 | 1.16 | [0.98, 1.38] | 0.091 |
| Anti-GLURP R2 (changing) | 1.23 | [1.08, 1.40] | 0.002 | 1.07 | [0.91, 1.27] | 0.410 |
| Anti-MSP3 (baseline) | 1.03 | [0.93, 1.14] | 0.565 | - | - | - |
| Anti-GLURP R0 (baseline) | 1.03 | [0.90, 1.17] | 0.709 | - | - | - |
| Anti-GLURP R2 (baseline) | 1.07 | [0.94, 1.21] | 0.302 | - | - | - |
| Month of birth |  |  |  |  |  |  |
| October | 1 | - | - | 1 | - | - |
| November | 2.01 | [0.68, 5.93] | 0.206 | - | - |  |
| December | 2.07 | [0.69, 6.25] | 0.195 | - | - | - |
| January | 2.61 | [0.84, 8.11] | 0.096 | - | - | - |
| EPI status (baseline) |  |  |  |  |  |  |
| Up to date | 1 | - | - | 1 | - | - |
| Not up to date | 1.16 | [0.68, 1.97] | 0.583 | - | - | - |
| Age mother (baseline) | 0.99 | [0.96, 1.03] | 0.910 | - | - | - |
| Gravidity status |  |  |  |  |  |  |
| Primigravidae | 1 | - | - | 1 | - | - |
| Multigravidae | 1.04 | [0.62, 1.73] | 0.889 | - | - | - |
| ITN use (pregnancy) |  |  |  |  |  |  |
| Yes | 1 | - | - | 1 | - | - |
| No | 1.28 | [0.74, 2.24] | 0.377 | 1.23 | [0.80, 1.88] | 0.348 |
| IPTp courses |  |  |  |  |  |  |
| 0 | 1 | - | - | 1 | - | - |
| 1 | 1.35 | [0.55, 3.32] | 0.517 | - | - | - |
| 2 | 0.74 | [0.30, 1.80] | 0.507 | - | - | - |
| 3 | 0.60 | [0.11, 3.37] | 0.566 | - | - | - |
| Education level (mother) |  |  |  |  |  |  |
| None | 1 | - | - | 1 | - | - |
| Primary | 1.43 | [0.94, 2.19] | 0.095 | 1.40 | [0.94, 2.09] | 0.093 |
| Secondary or above | 0.25 | [0.08, 0.72] | 0.011 | 0.24 | [0.06, 0.90] | 0.035 |
| Zone of residence |  |  |  |  |  |  |
| Rural | 1 | - | - | 1 | - | - |
| Urban | 0.61 | [0.33, 1.14] | 0.122 | - | - | - |
| Mixed | 1.38 | [0.84, 2.26] | 0.199 | - | - | - |
| Season |  |  |  |  |  |  |
| Dry season | 1 | - | - | 1 | - | - |
| Rains | 6.07 | [3.62, 10.15] | <0.001 | 4.57 | [2.59, 8.07] | <0.001 |
| Malaria Exposure index | 1.05 | [1.01, 1.10] | 0.012 | 1.03 | [0.99, 1.07] | <0.001 |
