# Supplementary material for: Plasmodium falciparum Malaria in Children Aged 0-2 Years: The Role of Foetal Haemoglobin and Maternal Antibodies to Two Asexual Malaria Vaccine Candidates (MSP3 and GLURP)
Source: PLoS One. 2014 Sep 19;9(9):e107965. doi: 10.1371/journal.pone.0107965 (PMC4169582; doi:10.1371/journal.pone.0107965)
Supplement: Table S6 — Multicollinearity diagnostics for continuous independent variables used in multivariable regression models. (DOCX) [file pone.0107965.s012.docx]

**Table S6**. Multicollinearity diagnostics for continuous independent variables used in multivariable regression models.

| **Variable** | **VIF** | **Square root (VIF)** | **Tolerance** | **R-Squared** |
| --- | --- | --- | --- | --- |
| Age | 1.06 | 1.03 | 0.9402 | 0.0598 |
| Anti-MSP3 (changing) | 1.31 | 1.15 | 0.7627 | 0.2373 |
| Anti-GLURP R0 (changing) | 1.48 | 1.22 | 0.6761 | 0.3239 |
| Anti-GLURP R2 (changing) | 1.52 | 1.23 | 0.6589 | 0.3411 |
| Fœtal Hb fraction (baseline) | 1.04 | 1.02 | 0.9611 | 0.0389 |
| MUAC | 1.02 | 1.01 | 0.9837 | 0.0163 |
| Malaria Exposure Index | 1.05 | 1.03 | 0.9514 | 0.0486 |
