# Supplementary material for: Plasmodium falciparum Malaria in Children Aged 0-2 Years: The Role of Foetal Haemoglobin and Maternal Antibodies to Two Asexual Malaria Vaccine Candidates (MSP3 and GLURP)
Source: PLoS One. 2014 Sep 19;9(9):e107965. doi: 10.1371/journal.pone.0107965 (PMC4169582; doi:10.1371/journal.pone.0107965)
Supplement: Table S7 — Testing for interactions between predictors in multivariable negative binomial regression model. (DOCX) [file pone.0107965.s013.docx]

**Table S7**. Testing for interactions between predictors in multivariable negative binomial regression model.

| **Predictor** | **IRR** | **p** | **95% CI** |
| --- | --- | --- | --- |
| Age | 1.13 | <0.001 | [1.08, 1.18] |
| Anti-MSP3 (changing) | 1.10 | 0.678 | [0.72, 1.68] |
| Anti-GLURP R0 (changing) | 1.25 | 0.36 | [0.77, 2.03] |
| Anti-GLURP R2 (changing) | 1.20 | 0.298 | [0.85, 1.69] |
| Foetal Hb fraction (baseline) | 0.98 | 0.011 | [0.97, 0.99] |
| Haemoglobin type |  |  |  |
| AA |  |  |  |
| AS^c^ | NA | - | - |
| AC | 1.25 | 0.386 | [0.75, 2.07] |
| CC | 0.38 | 0.045 | [0.14, 0.98] |
| MUAC (baseline) | 1.32 | 0.001 | [1.11, 1.58] |
| ITN use (pregnancy) |  |  |  |
| Yes |  |  |  |
| No | 1.31 | 0.322 | [0.77, 2.26] |
| Season |  |  |  |
| Dry season |  |  |  |
| Rains | 1.40 | 0.025 | [1.04, 1.87] |
| Malaria Exposure Index | 1.13 | 0.001 | [1.05, 1.21] |
| Number previous infections | 0.33 | <0.001 | [0.19, 0.60] |
| Month of birth*Age |  |  |  |
| November | 1.02 | 0.382 | [0.98, 1.07] |
| December | 1.02 | 0.427 | [0.98, 1.06] |
| January | 1.03 | 0.249 | [0.98, 1.07] |
| Anti-MSP3*Anti-GURP R0 | 0.98 | 0.591 | [0.91, 1.05] |
| Anti-MSP3*Anti-GURP R2 | 0.97 | 0.398 | [0.91, 1.04] |
| Anti-GURP R0*Anti-GURP R2 | 0.98 | 0.471 | [0.92, 1.04] |
| Anti-MSP3*Number previous infections | 1.03 | 0.533 | [0.93, 1.15] |
| Anti-GLURP R0*Number previous infections | 0.97 | 0.657 | [0.83, 1.13] |
| Anti-GLURP R2*Number previous infections | 1.11 | 0.108 | [0.98, 1.25] |
| Exposure Index*Anti-MSP3 | 0.98 | 0.04 | [0.96, 0.99] |
| Exposure Index*Anti-GLURP R0 | 0.99 | 0.989 | [0.98, 1.02] |
| Exposure Index*Anti-GLURP R2 | 1.004 | 0.585 | [0.99, 1.02] |

(*) Interaction term
